# Changes in olfaction and taste in patients hospitalized for COVID-19 and their relationship to patient evolution during hospitalization

**DOI:** 10.1016/j.bjorl.2021.11.002

**Published:** 2021-11-24

**Authors:** Pedro Antônio O.A. Gusmão, José Reinaldo C. Roveda, Ana Sophia M. Leite, Arnaldo S. Leite, Carolina C. Marinho

**Affiliations:** a Universidade Federal de Minas Gerais, Faculdade de Medicina, Belo Horizonte, MG, Brazil; b Faculdade Ciências Médicas de Minas Gerais, Belo Horizonte, MG, Brazil; c Universidade Federal de Minas Gerais, Faculdade de Medicina, Departamento de Clínica Médica, Belo Horizonte, MG, Brazil

**Keywords:** COVID-19, Sars-CoV-2, Olfactory disorders, Taste disorders

## Abstract

**Objectives:**

During the COVID-19 pandemic, several cases of changes in olfaction and taste associated with the infection have been reported. Therefore, otolaryngologists are frequently the first medical professionals sought by patients. The aim of this study was to evaluate the frequency of olfaction and taste disorders in patients hospitalized with COVID-19, and their association with other clinical manifestations and patient evolution during hospitalization.

**Methods:**

248 patients, admitted to three public hospitals in Belo Horizonte, Minas Gerais, Brazil, were prospectively included: Hospital das Clínicas da Universidade Federal de Minas Gerais (HC-UFMG), Hospital Júlia Kubitschek (HJK) and Hospital Eduardo de Menezes (HEM), who, upon admission to hospital, presented with Severe Acute Respiratory Syndrome due to COVID-19. Clinical and laboratory variables and outcomes during hospitalization were prospectively collected from the electronic medical records. The collection of sociodemographic and symptomatology data during the acute phase was carried out prospectively in electronic medical records and confirmed with the patients at a subsequent outpatient visit.

**Results:**

The most frequently reported symptoms were dyspnea (77.4%), cough (69.8%) and fever (55.2%). During the acute phase of the disease, 95 (38.3%) and 87 (35.1%) patients reported taste and olfaction disorders, respectively. There was a lower prevalence of dysosmia among patients with previous comorbidities (*p* < 0.05). Both symptoms were associated with less need for intensive care admission (*p* = 0.001 for dysgeusia and *p* = 0.021 for dysosmia) and a negative correlation with length of hospital stay (dysosmia: r = −0.175, *p* < 0.05; dysgeusia: r = −0.29, *p* < 0.001) and length of stay in the ICU (dysosmia: r = −0.136, *p* < 0.05; dysgeusia: r = −0.215, *p* < 0.05). The absence of taste disorders was also associated with a greater need for mechanical ventilation (*p* < 0.001).

**Conclusion:**

Changes in taste and olfaction were reported by a large number of patients in the acute phase of COVID-19. In this study, both were markers of better clinical patient evolution.

**Level of evidence:**

1B.

## Highlights

• A significant number of the patients had olfaction and taste disorders.
• Olfaction and taste disorders were markers of better clinical evolution.
• Predictors of better evolution can help to decongest the health system.

## Introduction

In November 2019, a new respiratory coronavirus, named SARS-CoV-2, responsible for an outbreak of pneumonia in Wuhan, China, was identified. Since then, the infection has spread around the world and was considered a pandemic by the World Health Organization (WHO) in March 2020. SARS-CoV-2 has a high infectivity rate, and the disease caused by it, called COVID-19, has the potential to result in significant complications. Due to these characteristics, the disease constitutes a burden to the public and private healthcare systems worldwide. One year after the index case, Brazil registered more than 11 million infected people, with 270,000 deaths up to March 12, 2021.^1^

Because it is a viral infection that causes a high frequency of respiratory and upper airway symptoms, otolaryngologists are often the first medical professionals sought by the patients. The main symptoms are cough, sore throat and dyspnea. Rhinorrhea, taste disorders (TD), and olfaction disorders (OD) are less common manifestations.^2^

Several cases of changes in olfaction and taste associated with COVID-19 were reported during the pandemic, symptoms that are common to other upper airway infections, such as those caused by the influenza, parainfluenza and respiratory syncytial virus. These diseases can affect olfaction through the swelling of the nasal mucosa secondary to a local inflammatory response or through direct nerve damage.^2^

Although the pathophysiology of OD and TD in COVID-19 is not fully elucidated, damage to the olfactory neuroepithelium is believed to be the predominant mechanism.^2^ Regarding the TDs, there are different hypotheses that aim to explain the loss of taste. The first is related to the change in olfaction itself, as the taste sensation depends on the retronasal stimulation pathway.^3^

Given the above, the aim of this study was to describe changes in olfaction and taste in patients hospitalized with COVID-19. The association between these alterations and other clinical manifestations and patient evolution during hospitalization was also evaluated.

## Methods

The study prospectively included 248 patients admitted to three public hospitals in Belo Horizonte, state of Minas Gerais, Brazil: Hospital das Clínicas da Universidade Federal de Minas Gerais (HC-UFMG), Hospital Júlia Kubitschek (HJK) and Hospital Eduardo de Menezes (HEM). Patients aged 18 years or over who, when admitted to the hospital, had Severe Acute Respiratory Syndrome (SARS) due to COVID-19 were included. SARS was characterized as defined by the Brazilian Ministry of Health: hospitalized individual, with fever and cough or sore throat, associated with dyspnea, chest tightness, or O_2_ saturation <95%.^4^ Confirmation of the disease was made by detection of the SARS-CoV-2 by Reverse-Transcriptase Polymerase Chain Reaction (RT-PCR). Patients were included in the study during hospital stay and after giving formal consent. Patients referred to palliative care were excluded. Patients who died during hospitalization and had already consented to participate in the study were included in the analysis. The study received the ethical approval of the National Research Ethics Commission, (CONEP, *Comissão Nacional de Ética em Pesquisa*) under number 30753020.1.1001.5149. The study was funded by the Office of the Dean of Research at Universidade Federal de Minas Gerais (PRPq-UFMG).

The collection of sociodemographic data, clinical variables at hospital admission, review of symptoms during the acute phase, including TD and OD, laboratory data and outcomes during hospitalization was performed retrospectively, by consulting the electronic medical record systems. Data were obtained by consulting the electronic medical record systems of the respective hospitals. Clinical and symptom data were confirmed by trained interviewers, according to a questionnaire standardized per research protocol with all patients who attended the outpatient consultation 45 days after hospital discharge, carried out at the Pulmonology and Internal Medicine Services of HC-UFMG. A validated test for objective analysis of olfaction and taste disorders was not performed. No protocol was established for the initial anamnesis in the emergency departments involved in the study.

The following variables were analyzed: age; self-declared ethnicity; gender; level of schooling; ICU admission; need for Mechanical Ventilation (MV); presence of comorbidities; initial symptoms (fever; cough; odynophagia; dyspnea; rhinorrhea; OD; TD; abdominal pain; diarrhea; other complaints). Data were stored on the REDCap platform (Vanderbilt University, USA) and analyzed by IBM SPSS 26 (IBM Corp.; Armonk, NY). Categorical variables were described as frequencies, and continuous variables, as median and interquartile range (IQR). Pearson’s Chi-Square test was used to compare categorical variables and Spearman’s test was used to correlate continuous variables. Statistical significance was defined as a *p*-value <0.05.

## Results

Of the 248 study participants, 127 (51.2%) were men, with a median age of 60.5 (47.2–69) years. Regarding the previous medical history, 216 (87.1%) had comorbidities, with Systemic Arterial Hypertension (SAH) and obesity being the most prevalent ones, in 146 (58.9%) and 80 (33.6%) participants, respectively.

At hospital admission, during the acute phase of COVID-19, the most common symptoms were dyspnea (77.4%), cough (69.8%) and fever (55.2%). Among the otorhinolaryngological complaints, TD stood out in 95 (38.3%) and OD, reported by 87 (35.1%) participants. The simultaneous occurrence of TD and OD were reported by 70 (28.2%) patients.

During hospital stay, 102 (41.1%) required intensive care, and 33 (13.3%) required MV. Four (1.6%) patients died.

Table 1 shows the sociodemographic characteristics and past medical history, clinical manifestations in the acute phase and hospital outcomes. The prevalence rates were stratified according to the presence or absence of OD and TD.

**Table 1:** Sociodemographic and clinical characteristics.

Clinical and demographic variable		Total n (%)	Olfaction disorders — n (%)		Taste disorders — n (%)		Taste and olfaction disorders — n (%)	
			Yes	No	Yes	No	Yes	No
Sociodemographic data								
Gender	Male	127 (51.2%)	38 (43.7%)	89 (55.3%)	44 (46.3%)	83 (54.2%)	29 (41.4%)	98 (55,1%)
	Female	121 (48.8%)	49 (56.3%)	72 (44.7%)	51 (53.7%)	70 (45.8%)	41 (58.6%)	80 (44,9%)
Self-declared ethnicity (n = 238)	Brown	127 (51.2%)	43 (49.4%)	84 (52.2%)	46 (48.4%)	81 (52.9%)	33 (47.1%)	94 (52,8%)
	White	60 (24.2%)	25 (28.7%)	35 (21.7%)	25 (26.3%)	35 (22.9%)	20 (28.6%)	40 (22,5%)
	Black	50 (20.2%)	18 (20.7%)	32 (19.9%)	23 (24.2%)	27 (17.6%)	16 (22.9%)	34 (19,1%)
	Yellow	1 (0.4%)	1(1.1%)	0 (0.0%)	1 (1.1%)	0 (0%)	1 (1.4%)	0 (0%)
Level of schooling (n = 203)	None	11 (5.4%)	3 (3.9%)	8 (6.3%)	3 (3.2%)	8 (5.2%)	3 (4.6%)	8 (5,8%)
	Elementary school	102 (50.2%)	37 (48.1%)	65 (51.6%)	41 (52.1%)	61 (47.7%)	31 (47.7%)	71 (51,4%)
	High school	62 (30.5%)	26 (33.8%)	36 (28.6%)	28 (32.6%)	34 (29.1%)	21 (32.3%)	41 (29,7%)
	Higher education	21 (10.3%)	7 (9.1%)	14 (11.1%)	10 (11.6%)	11 (9.4%)	7 (10.8%)	14 (10,1%)
	Postgraduation	7 (3.4%)	4 (5.2%)	3 (2.4%)	4 (4.7%)	3 (2.6%)	3 (4.6%)	4 (2,9%)
Past medical history								
Presence of comorbidities		216 (87,1%)	72 (82.8%)	144 (89.4%)	77 (81.1%)	139 (90.8%)	56 (80%)	160 (89.9%)
Systemic arterial hypertension		146 (58,9%)	46 (53.5%)	100 (62.1%)	51 (54.3%)	95 (62.1%)	38 (55.1%)	108 (60.7%)
Obesity (n = 238)		80 (33,6%)	37 (46.3%)	43 (53.8%)	37 (35.4%)	43 (53.8%)	31 (45.6%)	49 (28.8%)
Diabetes mellitus		71 (28,6%)	21 (31.1%)	50 (24.1%)	19 (20%)	52 (34%)	13 (18.6%)	58 (32.6%)
Smoking		61 (24,6%)	21 (25.3%)	40 (24.4%)	20 (21.1%)	41 (27.5%)	17 (24.3%)	44 (25.3%)
Asthma		22 (8,9%)	13 (8.1%)	9 (10.5%)	8 (8.5%)	14 (9.2%)	5 (7.2%)	17 (9.6%)
COPD		18 (7,2%)	15 (9.3%)	3 (3.5%)	2 (2.2%)	16 (10.5%)	2 (2.9%)	16 (9%)
Use of ACEIs, ARBs or NSAIDs		114 (46%)	40 (46%)	74 (46.8%)	41 (43.2%)	73 (48.7%)	33 (47.1%)	81 (46.3%
Clinical manifestations in the acute phase								
Dyspnea		192 (77.4%)	71 (81.6%)	121 (75.2%)	76 (80%)	116 (75.8%)	56 (80%)	136 (76.4%)
Cough		173 (69.8%)	109 (67.7%)	64 (73.6%)	72 (75.8%)	101 (66%)	53 (75.7%)	120 (67.4%)
Fever		137 (55.2%)	57 (65.5%)	80 (49.7%)	60 (63.2%)	77 (50.3%)	45 (64.3%)	92 (51.7%)
Myalgia		113 (45.6%)	57 (65.5%)	56 (34.8%)	57 (60%)	56 (36.6%)	47 (67.1%)	66 (37.1%)
Taste disorders		95 (38.3%)	70 (80.5%)	25 (15.5%)	–	–	–	–
Olfaction disorders		87 (35.%1)	–	–	70 (80.5%)	17 (11.1%)	–	–
Diarrhea		59 (23.8%)	28 (32.2%)	31 (19.3%)	30 (31.6%)	29 (19%)	26 (37.1%)	33 (18.5%)
Rhinorrhea		48 (19.4%)	21 (24.1%)	27 (16.8%)	27 (28.4%)	21 (13.7%)	20 (28.6%)	28 (15.7%)
Sore throat		39 (15.7%)	16 (18.4%)	23 (14.3%)	22 (23.2%)	17 (11.1%)	15 (21.4%)	24 (13.5%)
Abdominal pain		26 (10.5%)	10 (11.5%)	16 (9.9%)	9 (9.5%)	17 (11.1%)	9 (12.9%)	17 (9.6%)
Hospital outcomes								
ICU admission		102 (41.1%)	27 (31%)	75 (46.6%)	26 (27.4%)	76 (49.7%)	21 (30%)	81 (45.5%)
Need for mechanical ventilation		33 (13.3%)	7 (8%)	26 (16.1%)	4 (4.2%)	29 (19%)	4 (5.7%)	29 (16.3%)
Length of hospital stay (days)^a^		10 (7–17)	9 (7–14)	12(7.75–18.5)	8 (6–20)	12 (8–13)	9 (7–14.25)	11 (1–18)
Length of ICU stay (days)^a^		0 (0–4)	0 (0–3)	0 (0–5)	0 (0–2)	0 (0–6)	0 (0–3)	0 (0–5)
Death		4 (1.6)					0	4 (2.2%)

COPD, chronic obstructive pulmonary disease; ACEIs, angiotensin-converting enzyme inhibitors; ARBs, angiotensin-receptor blockers; NSAIDs, non-steroidal anti-inflammatory drugs.

a Median (interquartile range).

Table 2 shows the comparison between patients with TD and/or OD and those without any of these two disorders. There was no association between either of the disorders with self-reported ethnicity, age or level of schooling. Fever, myalgia and diarrhea were associated with both disorders, with the exception of the TD + OD group, which did not show an association with fever. Moreover, rhinorrhea was associated with TD and TD + OD, while odynophagia was associated only with TD. A lower frequency of TD was observed in patients with previous comorbidities (Odds Ratio [OR]: 0.431; Confidence Interval [95% CI]: 0.203–0.914; *p* < 0.05), and the TD + OD group showed a similar association (Odds Ratio [OR]: 0.450; Confidence Interval [95% CI]: 0.210–0.964; *p* < 0.05). The need for admission to the intensive care unit (ICU) was associated with a lower frequency of TD (OR = 0.382; 95% CI: 0.22–0.663; *p* = 0.001), of OD (OR = 0.516; 95% CI: 0.298–0.894; *p* < 0.021) and TD + OD (OR = 0.513; 95% CI: 0.284–0.926; *p* < 0.05). Similarly, the absence of TD was also associated with greater need for MV (OR = 0.188; 95% CI: 0.064–0.553; *p* = 0.001), and the same association was observed in the TD + OD group (OR = 0.311; 95% CI: 0.105–0.921; *p* < 0.05). The length of hospital stay showed a weak but significant negative correlation with the absence of TD, OD and TD + OD. The length of stay in the ICU showed a weak negative correlation when the groups OD and TD were analyzed separately, but no statistical significance was observed in the TD + OD group.

**Table 2:** Association between demographic and clinical variables in the acute phase and olfaction and taste disorders.

Clinical and demographic variables		Olfaction disorders		Taste disorders		Taste and olfaction disorders	
		OR (95% CI)	*p* Value	OR (95% CI)	*p* Value	OR (95% CI)	*p* Value
Gender	Male	0.627 (0.371–1.061)	0.085	0.728 (0.435–1.216)	0.242	0.577 (0.330–1.011)	0.053
	Female	0.627 (0.371–1.061)	0.085	0.728 (0.435–1.216)	0.242	0.577 (0.330–1.011)	0.053
Age (years)	20–39	0.741 (0.337–1.631)	0.456	0.632 (0.288–1.389)	0.251	0.903 (0.399–2.045)	0.807
	40–59	1.742 (1.012–2.998)	0.044	1.745 (1.022–2.982)	0.041	1.299 (0.732–2.307)	0.371
	60–79	0.753 (0.444–1.277)	0.292	0.783 (0.467–1.315)	0.355	0.898 (0.514–1.569)	0.706
	80–100	0.694 (0.239–2.016)	0.5	0.792 (0.287–2.186)	0.652	0.710 (0.225–2.236)	0.577
Comorbidities		0.567 (0.268–1.199)	0.165	0.431 (0.203–0.914)	0.032	0.450 (0.210–0.964)	0.037
Taste disorders		22.4 (11.345–44.226)	<0.001	–	–	–	–
Olfaction disorders		–	–	22.4 (11.345–44.226)	<0.001	–	–
Cough		1.327 (0.743–2.37)	0.386	1.612 (0.906–2.868)	0.119	1.507 (0.803–2.829)	0.2
Dyspnea		1.467 (0.766–2.809)	0.269	1.276 (0.683–2.382)	0.533	1.235 (0.626–2.439)	0.542
Fever		1.924 (1.122–3.299)	0.023	1.692 (1.002–2.856)	0.05	1.683 (0.951–2.977)	0.072
Myalgia		3.563 (2.059–6.164)	<0.001	2.598 (1.535–4.397)	<0.001	3.468 (1.933–6.219)	<0.001
Diarrhea		1.99 (1.096–3.614)	0.029	1.913 (1.092–3.568)	0.031	2.596 (1.404–4.801)	0.002
Rhinorrhea		1.579 (0.831–3.001)	0.18	2.496 (1.315–4.738)	0.005	2.143 (1.111–4.134)	0.021
Odynophagia		1.352 (0.672–2.721)	0.465	2.411 (1.205–4.825)	0.013	1.750 (0.856–3.577)	0.122
Abdominal pain		1.177 (0.51–2.718)	0.828	0.837 (0.357–1.962)	0.832	1.397 (0.591–3.302)	0.444
Need for ICU admission		0.516 (0.298–0.894)	0.021	0.382 (0.22–0.663)	0.001	0.513 (0.284–0.926)	0.026
Mechanical ventilation		0.454 (0.189–1.094)	0.08	0.188 (0.064–0.553)	0.001	0.311 (0.105–0.921)	0.027
		*r*^a^	*p*	*r*^a^	*p*	*r*^a^	*p*
Hospital length of stay		−0.175	0.006	−0.136	0.033	−0.139	0.03
Time in the intensive care unit		−0.29	<0.001	−0.215	0.001	−0.122	0.057

a Spearman’s correlation.

## Discussion

The new coronavirus pandemic has become a burden to public and private healthcare systems worldwide.^5^, ^6^ During the pandemic, cases of changes in olfaction and taste associated with COVID-19 infection have been reported. In this study, the frequency of OD and TD was 35.1% and 38.3%, respectively. The presence of TD was inversely associated with the need for ICU admission (OR = 0.382; 95% CI 0.22–0.663), MV (OR = 0.188; 95% CI 0.064–0.533) and with the presence of previous comorbidities (OR = 0.431; 95% CI 0.230–0.914), while the presence of OD showed an inverse association with ICU admission (OR = 0.516; 95% CI 0.298–0.894). Both disorders were negatively correlated with length of hospital stay (OD: r −0.175, *p* < 0.05; TD: r −0.29, *p* < 0.001) and length of ICU stay (OD: r −0.136, *p* < 0.05; TD: r −0.215, *p* < 0.05). When analyzing the grouped olfaction and taste disorders, there was a change in the outcome length of ICU stay, which was not statistically significant. Among the other preemptive severity factors, no difference was observed in the pooled analysis.

The identification of better prognosis predictors can help in patient stratification and to decongest hospitals and ICU beds in the presence of health service overload. Moreover, the association of TD and OD with milder clinical pictures has epidemiological implications, since in context of scarcity of tests, these patients are less likely to be tested. Attention to these symptoms can increase the suspicion of infection in oligosymptomatic cases and contribute to reduced transmission by these individuals.^7^

The frequency of TD and OD varies widely in the literature. The meta-analysis carried out by Agyeman et al.,^7^ consisting of 24 studies and including 8438 patients, found a frequency of 41% for OD and 38.2% for TD in COVID-19. However, the severity of the included patients was not described in the study. Although comparable to the frequencies reported herein (38% and 35%, respectively), the present population only included patients hospitalized with the severe forms of COVID-19. Additionally, the inverse relationship between the frequency of OD and age described by the authors was not detected in the present study. When analyzing individual studies, Lechien et al.^8^ reported the occurrence of 85.6% and 88.8% of OD and TD, respectively, in patients with the mild to moderate form of COVID-19. Chen et al. found a frequency of 3.4% (TD) and 3.4% (OD) in patients with the severe form of the disease.^9^ A study using a self-administered questionnaire via the internet^10^ reported a frequency of 59.2% for olfaction and/or taste disorders among outpatients. These frequencies described by other authors in specific severity groups are compatible with the association of TD and OD with lower severity and better hospital outcomes found in the present study.

Few published studies have analyzed the relationship between TD and OD and the clinical evolution of patients with COVID-19. The study by Yan et al. demonstrated that patients with OD were ten times less likely to be hospitalized (OR = 0.09; 95% CI 0.01–0.74) compared to those without loss of olfaction.^11^ Izquierdo-Domínguez et al. reported that the presence of OD had an inverse relationship with the need for hospitalization, CRP levels and age. TDs were inversely associated with need for hospitalization and age.^12^ Up to now, the present study is the first to analyze the relationship between olfaction-taste disorders and the need for MV and ICU admission. A limitation of this approach is the possible presence of recall bias: patients with more severe symptoms, such as dyspnea, could neglect milder symptoms, such as dysosmia and dysgeusia.

The explanation for the different prevalence rates of OD and TD among different populations remains to be elucidated. The Angiotensin-2 Converting Enzyme (ACE2) is known to act as a receptor for the new coronavirus. The binding of the enzyme and the virus seems to be related to the disease pathophysiology. It is believed that the presence of this enzyme in the olfactory epithelium, especially in the olfactory bulb, can turn the respiratory epithelium into a viral reservoir,^2^, ^13^, ^14^ as well as a facilitator for SARS-CoV-2 brain infections.^14^, ^15^, ^16^, ^17^

Recent studies suggest that ACE2 could be specific for some populations. In a recent study by Cao et al.,^18^ 32 variants of ACE2 were found. Li et al.^19^ demonstrated that some ACE2 variants could reduce the association between human ACE2 and the S protein of SARS-CoV-1. Thus, the difference in ACE2 expression levels in different tissues could explain some of the differences related to COVID-19 susceptibility, symptoms and outcomes. Although a gap remains in the knowledge of the disease pathophysiology, a hypothesis that could explain the association between OD and TD and the clinical course of patients with COVID-19 would be that different expressions of ACE2 could be determinants of the immune response to SARS-CoV-2 infection: the preferential binding to the olfactory neuroepithelium could lead to an infection located predominantly in the upper airways and, consequently, to a less fulminant infection, allowing the earlier development of antibodies. In this study, the presence of TD and OD was associated with shorter hospital and ICU stays, possible indicators of less severe disease.

Regarding the TDs, there are some hypotheses that aim to explain the loss of taste. The first is related to the change in olfaction itself, as the taste sensation depends on the retronasal stimulation pathway.^3^ On the other hand, some authors suggest that the damage is due to the presence of ACE2 receptors in the oral cavity mucosa and tongue epithelial cells.^20^, ^21^

A limitation of this study is the use of subjective assessment to measure OD and TD through self-reports, without checking the degree of loss of the respective senses. There was no systematic assessment by an otorhinolaryngologist in the first outpatient follow-up meeting. Olfaction-taste disorders can be assessed through questionnaires (validated or not), self-reporting, medical record information, or objective methods, such as chemosensory testing.^22^ In the meta-analysis performed by Agyeman et al.,^7^ there was a statistical difference between subjectively and objectively assessed OD (coefficient = 2.33; 95% CI 0.57–4.09; *p* = 0.01); this difference did not occur for the TDs. Hannum et al.^22^ performed a meta-analysis specifically seeking to assess whether there is a difference between the objective and subjective evaluations of OD, finding a statistical difference (76.7%, 95% CI 61.4–89.2; and 44.4%, 95% CI 32.2–57.0; respectively). It is noteworthy that few studies used objective methods due to the greater assessment complexity and the inherent risks in the context of the pandemic.

More studies are needed to better describe the identified associations, preferably using objective methods for the detection of disorders. It is also important to study the long-term prognosis, as well as possible sequelae in patients who had OD and TD in the acute phase of COVID-19 infection.

## Conclusion

In this study, OD were associated with a lower risk of hospitalization and shorter ICU stay, while TD were associated with a lower risk of ICU admission and need for MV, in addition to shorter hospital and ICU stay. These data can be useful as a screening tool, especially in countries with fewer resources in the health area.

The study was funded by the Dean of Research at Universidade Federal de Minas Gerais (PRPq-UFMG). The study received the ethical approval of the National Research Ethics Commission (CONEP) under number 30753020.1.1001.5149.

## Conflicts of interest

The authors declare no conflicts of interest.

All authors participated equally in data collection, statistical analysis and article development. The authors declare no conflicts of interest.
